# Epac‐2 ameliorates spontaneous colitis in *Il‐10* ^−/−^ mice by protecting the intestinal barrier and suppressing NF‐κB/MAPK signalling

**DOI:** 10.1111/jcmm.17077

**Published:** 2021-12-03

**Authors:** Xue Song, Hexin Wen, Lugen Zuo, Zhijun Geng, Jing Nian, Luyao Wang, Yifan Jiang, Jing Tao, Zihan Zhu, Xiaopei Wu, Zhikun Wang, Xiaofeng Zhang, Liang Yu, Hao Zhao, Ping Xiang, Jing Li, Lin Shen, Jianguo Hu

**Affiliations:** ^1^ Department of Central Laboratory First Affiliated Hospital of Bengbu Medical College Bengbu China; ^2^ Anhui Key Laboratory of Tissue Transplantation Bengbu Medical College Bengbu China; ^3^ Department of Gastrointestinal Surgery First Affiliated Hospital of Bengbu Medical College Bengbu China; ^4^ Department of Imaging Second Affiliated Hospital of Bengbu Medical College Bengbu China; ^5^ Department of Clinical Medicine Bengbu Medical College Bengbu China; ^6^ Department of Clinical Laboratory First Affiliated Hospital of Bengbu Medical College Bengbu China

**Keywords:** Crohn's disease, Epac‐2, intestinal barrier, macrophage, TJ protein

## Abstract

Intestinal barrier dysfunction and intestinal inflammation interact in the progression of Crohn's disease (CD). A recent study indicated that Epac‐2 protected the intestinal barrier and had anti‐inflammatory effects. The present study examined the function of Epac‐2 in CD‐like colitis. Interleukin‐10 gene knockout (*Il*‐*10* ^−/−^) mice exhibit significant spontaneous enteritis and were used as the CD model. These mice were treated with Epac‐2 agonists (Me‐cAMP) or Epac‐2 antagonists (HJC‐0350) or were fed normally (control), and colitis and intestinal barrier structure and function were compared. A Caco‐2 and RAW 264.7 cell co‐culture system were used to analyse the effects of Epac‐2 on the cross‐talk between intestinal epithelial cells and inflammatory cells. Epac‐2 activation significantly ameliorated colitis in mice, which was indicated by reductions in the colitis inflammation score, the expression of inflammatory factors and intestinal permeability. Epac‐2 activation also decreased Caco‐2 cell permeability in an LPS‐induced cell co‐culture system. Epac‐2 activation significantly suppressed nuclear factor (NF)‐κB/mitogen‐activated protein kinase (MAPK) signalling in vivo and in vitro. Epac‐2 may be a therapeutic target for CD based on its anti‐inflammatory functions and protective effects on the intestinal barrier.

## 1 INTRODUCTION

Crohn's disease (CD) is a chronic, recurrent and non‐specific transmural type of inflammation commonly found in the digestive tract.^1^ CD is characterized by abdominal pain, diarrhoea, anaemia and associated malnutrition, which detrimentally impact the patient's quality of life.^2^ The incidence of CD has increased steadily with improvements in the standard of living.^3^ The predisposing factors and specific pathological mechanisms of CD are not fully understood. However, imbalances in immune homeostasis, intestinal barrier dysfunction and bacteria are major contributors to the pathogenesis of CD.^4^

Improvements in intestinal barrier function positively impact the treatment and recovery of CD.^5^ When the intestinal barrier is disrupted, bacteria enter the human body through the intestinal mucosa, which leads to local or systemic inflammation.^6^ The structure of the epithelial barrier is essential for resisting microbial entry and maintaining homeostasis, and a major goal in the treatment of CD is to promote intestinal epithelial repair.^7^

The mucosal barrier includes the epithelial barrier, which hinders the spread of macromolecular substances.^8^ Epithelial cells in the intestinal barrier connect cell boundaries via tight junctions (TJs), and the functions of the intestinal barrier are primarily performed by TJs, the mucus layer and antibiotic peptides.^9^ Many bacterial pathogens destroy TJs by inhibiting the expression of zonula occludens‐1 (ZO‐1) and occludin, and the destruction of TJ structure and function in epithelial cells leads to the loss of barrier integrity.^10^ Inflammation induces the pathophysiological mechanism of intestinal injury.^11^ The activation of cytokines (interleukin (IL)‐17A, tumour necrosis factor‐alpha (TNF‐α) and IL‐1β) may inhibit the expression of TJ protein and lead to the destruction of intestinal barrier function.^12^

The exchange protein Epac‐2 is activated by cAMP, and it is the link between Ras and Rap‐1. Epac‐2 is the major Epac isoform expressed in the gut.^13^, ^14^ Epac‐2 activation upregulates the expression of Rap‐1.^15^ Recent studies showed that Rap‐1 robustly regulated the functions of adherens junctions (AJs) and TJs in vitro and in vivo and improved the function of the epithelial barrier by promoting the expression of ZO‐1 and occludin.^16^ Epac‐2 activation reverses the attenuation of TJ proteins and inhibits macrophage production of proinflammatory cytokines via Rap‐1 activation.^17^ Inhibition of Rap‐1 expression reduces the activity of macrophages,^18^ and the role of Rap‐1 in barrier restoration was further described. Anna and colleagues showed that Epac‐2 activation‐induced overexpression of Rap‐1 inhibited inflammation by attenuating p38 mitogen‐activated protein kinase (MAPK) and nuclear factor kappa B (NF‐κB) p65 signalling.^19^, ^20^ These data suggest that Epac‐2 plays key roles in regulating macrophage production of proinflammatory cytokines and maintaining the function of the intestinal barrier in CD patients.

The *Il*‐*10* ^−/−^ model is the closest animal model to the CD phenotype.^21^ We investigated the clinical results and effective mechanisms of Epac‐2 in *Il*‐*10* ^−/−^ mice treated with Me‐cAMP (an Epac‐2 agonist) or HJC‐0350 (an Epac‐2 antagonist). Colonic expression of Epac‐2 was abnormal in CD patients, and Epac‐2 activated the Rap‐1 pathway and inhibited the phosphorylation of NF‐κB/MAPK in macrophages, which increased the levels of ZO‐1 and occludin in intestinal epithelial cells. These results suggest that the targeting of Epac‐2 will improve the current treatment of CD.

## 2 MATERIALS AND METHODS

### 2.1 Patient specimen preparation

Intestinal specimens were collected from patients with CD (*n* = 12) who underwent intestinal resection, and uninjured bowel tissue was collected from colon cancer patients (control, *n* = 16), as described in Appendix S1.

### 2.2 Animals

Wild‐type (WT) and *Il*‐*10* ^−/−^ (15 weeks old) mice were purchased from the Jackson Laboratory of America. *Il*‐*10* ^−/−^ mice often develop CD‐like colitis when reared under normal conditions^22^ (Appendix S1).

### 2.3 Drug administration

*Il*‐*10* ^−/−^ mice with spontaneous colitis were divided into 3 groups (*n* = 10): Me‐cAMP‐treated (10 μl, 100 μmol/L, i.p. injection) group, the HJC‐0350 (0.5 mg/kg, i.p. injection) group and the *Il*‐*10* ^−/−^ group as a positive control.^23^, ^24^ The mice received treatment every 2 days until the end of 4 weeks. Wild‐type C57Bl/6 mice were used as the negative control (WT) group. After 4 weeks, the entire colon of each animal was harvested, and the length of the colon was measured^25^ (Appendix S1).

### 2.4 Colitis symptom assessment

Mice in all groups were evaluated for the extent of colitis using the disease activity index (DAI) score once weekly as previously described.^6^ The scoring interval for DAI was 0–5 (Appendix S1).

### 2.5 Histopathological analysis

Colon tissue was fixed and scored based on the grade of intestinal inflammation.^26^ The scoring interval for intestinal inflammation in the mice was 0 to 4 (Appendix S1).

### 2.6 ELISA

Protein levels in lysates extracted from frozen samples of colon tissue were measured using the following ELISA kits: IL‐1β (R&D Systems, Emeryville, Cat #: MLB00C), TNF‐α (R&D Systems, Emeryville, Cat #: MTA00B) and IL‐17A (R&D Systems, Emeryville, Cat #: M17AF0).

### 2.7 FITC‐dextran permeability assay

After completion of the therapeutic schedule, the mice were fasted under water restriction for 4 h then administered oral FITC‐dextran (Sigma, Cat #: F‐7250) as previously reported^27^ (Appendix S1).

### 2.8 Bacterial content

Bacteria were isolated from mesenteric lymph nodes (MLNs) and liver tissue using a previously described method^28^ (Appendix S1).

### 2.9 Cell culture and treatment

Caco‐2 and RAW 264.7 cell lines were obtained from the Shanghai Institutes for Biological Science (Shanghai, China). The cells were incubated for 24 h with (1) normal DMEM (control); (2) 1 µg/ml lipopolysaccharide (LPS); (3) 1 µg/ml LPS and 50 µmol/L Me‐cAMP; or (4) 1 µg/ml LPS and 0.3 µmol/L HJC‐0350^29^, ^30^ (Appendix S1).

### 2.10 Trans‐epithelial electric resistance (TEER) evaluation

Trans‐epithelial electric resistance was performed in a Transwell co‐culture system. RAW 264.7 cells were pre‐treated with 50 µmol/L Me‐cAMP or 0.3 µmol/L HJC‐0350 for 1 h then induced with 1 μg/ml LPS for 24 h (Appendix S1).

### 2.11 Cell permeability to FD4

After TEER measurement, cell permeability was evaluated using FD4 (Appendix S1).

### 2.12 Cell co‐culture assay

RAW 264.7 cells were pre‐treated with 50 µmol/L Me‐cAMP or 0.3 µmol/L HJC‐0350 for 1 h then incubated with 1 μg/ml LPS for 24 h. The cell supernatants were collected and centrifuged (12,000 *g*; 10 min), added to the corresponding Caco‐2 cells, and incubated for 24 h (Appendix S1).

### 2.13 Flow cytometry analysis

The apoptosis rates of Caco‐2 cells were determined and analysed using a FACSCalibur flow cytometer (Appendix S1).

### 2.14 Immunofluorescence and immunohistochemical analysis

Antibodies against ZO‐1 and occludin (1:100; Abcam) were used for immunofluorescence (IF) to observe the distribution of TJ proteins in the intestinal mucosa and Caco‐2 cells. Antibodies against Epac‐2 (1:100; Abcam) were used for immunohistochemistry (IHC) on human colonic tissue^31^ (Appendix S1).

### 2.15 Western blot analysis

Western blotting (WB) was performed as previously described to measure protein levels in tissues and cells.^6^ Primary antibodies against occludin, ZO‐1, Epac‐2, Rap‐1, NF‐κB p65 (p65), p‐NF‐κB p65 (p‐p65), p‐p38 MAPK (p‐p38), p38 MAPK (p38) and β‐actin were used (Abcam) at dilutions of 1:1,000. Densitometric analysis of protein band intensity was performed with ImageJ (National Institutes of Health, USA). The results are presented as the relative density of each experimental band with respect to the density of the β‐actin band normalized to the lowest mean value group.

### 2.16 Quantitative real‐time PCR (qRT‐PCR)

qRT‐PCR was performed as previously reported to measure the mRNA levels^27^ of TNF‐α, IL‐1β, IL‐17A and β‐actin in tissues. The sequence information for mouse gene‐specific primers is provided in the Appendix S1.

### 2.17 Statistical analysis

The collected experimental data were statistically analysed using SPSS 17.0 (SPSS Inc., Chicago, IL). Independent samples *t* tests were used to assess data from two groups, and chi‐squared tests were used to assess categorical data. Findings with P values less than 0.05 were statistically significant.

## 3 RESULTS

### 3.1 Epac‐2 expression was decreased in CD patients

**FIGURE 1:**
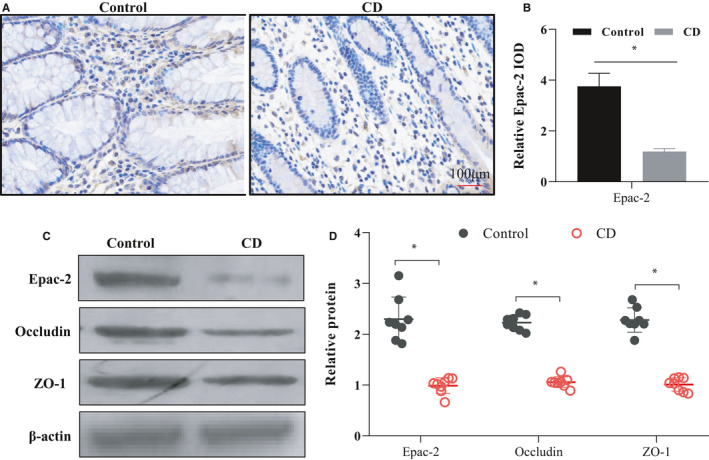
Epac‐2 expression was decreased in CD patients. (A, B) Immunohistochemistry was performed to analyse the expression of Epac‐2 in the colon of the CD group (*n* = 12) and control group (*n* = 16). The results are shown as the mean integrated optical density (IOD) ± SD. (C, D) Eight samples were randomly selected from CD patients and control patients for WB. The results revealed the expression of Epac‐2, occludin and ZO‐1 in the inflammatory foci of colon tissue from the CD group and uninflamed colon tissue from the control group. The relative protein is shown as fold change of protein expression in the CD group relative to the control group. IOD, integrated optical density, ^*^ *p* < 0.05, compared to the control group

Immunohistochemistry revealed that the expression of Epac‐2 decreased significantly in colon tissue of the CD group compared with the control group (Figure 1A,B). Western blotting analyses of Epac‐2, occludin and ZO‐1 were performed on 8 randomly selected samples from CD and control patients. The data revealed that the expression of Epac‐2, occludin and ZO‐1 was suppressed in the inflammatory foci of colon tissue from the CD group compared to uninflamed colon tissue from the control group (Figure 1C,D). This result indicates a connection between the decrease in Epac‐2 expression and the pathological process of CD.

### 3.2 Effects of Epac‐2 on colitis in *Il*‐*10* ^−/−^ mice

**FIGURE 2:**
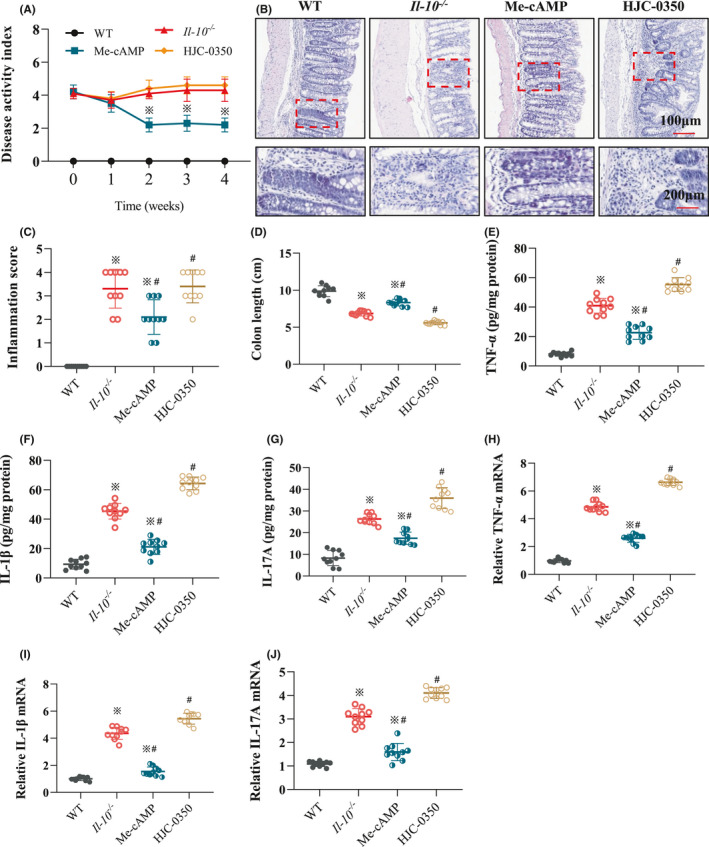
Effect of Epac‐2 on colitis in *Il*‐*10* ^−/−^ mice. (A) The DAI scores of mice in the WT group, *Il*‐*10* ^−/−^ group, Me‐cAMP group and HJC‐0350 group. The DAI was obtained using a 6‐point (0–5) scale. Each mouse was evaluated once weekly. (B) Haematoxylin and eosin (H&E) staining revealed the histological manifestations of colonic tissues in each group after 4 weeks of treatment. The magnifications are described below the images. (C) The inflammatory scores revealed the values of intestinal tissues in each group after treatment. (D) The colon lengths of mice in each group were measured and recorded after treatment completion. (E–G) The protein levels of TNF‐α, IL‐1β and IL‐17A in colons from mice in each group. (H–J) The mRNA levels of cytokines in colons from mice in each group. *Il*‐*10* ^−/−^ mice with spontaneous colitis were divided into 3 groups: the Me‐cAMP group, the HJC‐0350 group and the *Il*‐*10* ^−/−^ group (positive control). WT C57Bl/6 mice were used as the negative control (WT) group. The experiments were performed 3 independent times (*n* = 10), and the most typical result is shown. The relative mRNA is shown as the fold change of mRNA levels in the Me‐cAMP group, the HJC‐0350 group and the *Il*‐*10* ^−/−^ group relative to the WT group. The data are presented as the means ± SD (^※^ *p* < 0.05, compared to the WT group; ^#^ *p* < 0.05, compared to the *Il*‐*10* ^−/−^ group)

The DAI scores indicated lower values in the Me‐cAMP group than the *Il*‐*10* ^−/−^ and HJC‐0350 groups (Figure 2A). The inflammatory scores showed that the values of intestinal tissues in the Me‐cAMP group were lower than those in the *Il*‐*10* ^−/−^ and HJC‐0350 groups, but the values in the *Il*‐*10* ^−/−^ group score were lower than those in the HJC‐0350 group (Figure 2B,C). In contrast, the colon length in the Me‐cAMP group was longer than that in the *Il*‐*10* ^−/−^ and HJC‐0350 groups, but the length in the HJC‐0350 group was less than that in the *Il*‐*10* ^−/−^ group (Figure 2D). Compared with those of the *Il*‐*10* ^−/−^ group, the levels of cytokines (TNF‐α, IL‐1β and IL‐17A) in the Me‐cAMP group were remarkably downregulated, but the expression in the HJC‐0350 group was enhanced (Figure 2E–G). The qRT‐PCR data also revealed that the mRNA levels of these cytokines in the Me‐cAMP group were downregulated, but the levels in the HJC‐0350 group were increased (Figure 2H–J). Our data suggest that Epac‐2 activation decreases CD‐like colitis and that the attenuation of Epac‐2 activation in *Il*‐*10* ^−/−^ mice exacerbated the condition, does not affect normal WT mice (Figure S1).

### 3.3 Epac‐2 activation improved TJ protein distribution and reduced intestinal permeability in *Il*‐*10* ^−/−^ mice

**FIGURE 3:**
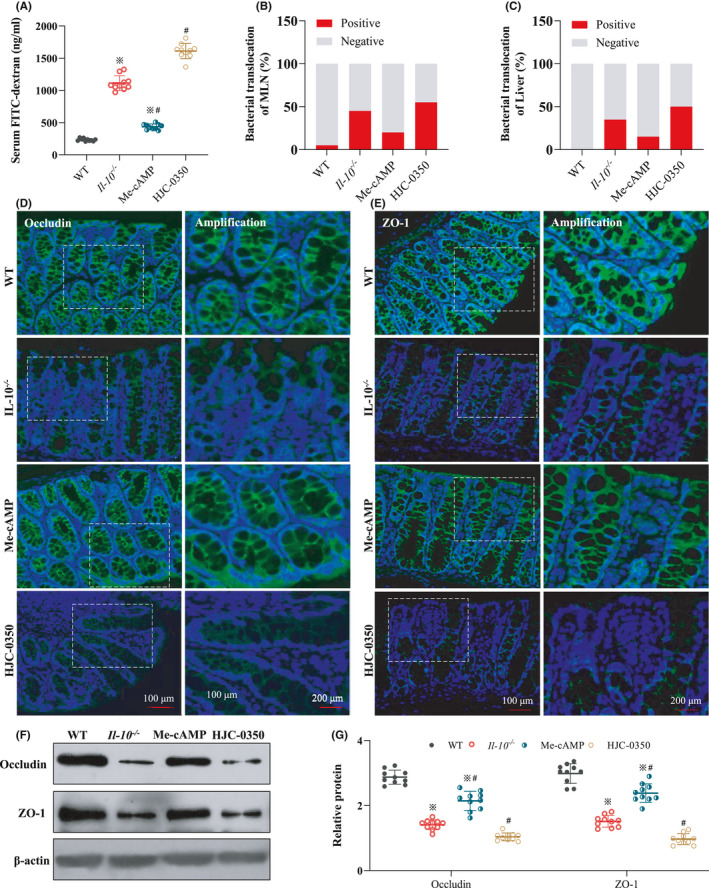
Epac‐2 activation improves the levels of TJ proteins and weakens intestinal permeability in *Il*‐*10* ^−/−^ mice. (A) The levels of serum FITC in the WT group, *Il*‐*10* ^−/−^ group, Me‐cAMP group and HJC‐0350 group. (B, C) The rate of bacterial translocation in the MLN and liver was evaluated using bacterial cultures. (D, E) Immunofluorescence analysis revealed the distribution of occludin (green) and ZO‐1 (green) in the intestinal mucosa of mice. DAPI (blue) was used to stain nuclei. (F, G) The WB results revealed the expression of Epac‐2, occludin and ZO‐1 in the intestinal mucosa of mice. *Il*‐*10* ^−/−^ mice with spontaneous colitis were divided into 3 groups: the Me‐cAMP group, the HJC‐0350 group and the *Il*‐*10* ^−/−^ group (positive control). WT C57Bl/6 mice were used as the negative control (WT) group. The experiments were performed 3 independent times (*n* = 10), and the most typical result is shown. The relative protein is shown as fold change of protein expression in the Me‐cAMP group, the WT group and the *Il*‐*10* ^−/−^ group relative to the HJC‐0350 group. The data are presented as the means ± SD (^※^ *p* < 0.05, compared to the WT group; ^#^ *p* < 0.05, compared to the *Il*‐*10* ^−/−^ group)

The results showed that the level of serum glucan binding was lower in the Me‐cAMP group than in the *Il*‐*10* ^−/−^ group but similar to that in WT mice. The level of serum glucan binding in the HJC‐0350 mice was higher than that in the *Il*‐*10* ^−/−^ mice (Figure 3A). Bacteria were isolated and cultured from mouse MLNs and liver tissues using sterile culture techniques. In the Me‐cAMP group, the chance of bacterial translocation to the liver and MLN was lower in the Me‐cAMP group than the *Il*‐*10* ^−/−^ group but similar to that in the WT group. However, the probability of bacterial transfer in the Me‐cAMP group exceeded that in the *Il*‐*10* ^−/−^ group (Figure 3B,C). Immunofluorescence and WB were used to measure occludin and ZO‐1 protein levels in mouse colons. The protein levels in the Me‐cAMP group were dramatically upregulated compared to those in the *Il*‐*10* ^−/−^ group. Nevertheless, the protein levels in the HJC‐0350 group and WT group were very similar (Figure 3D,E). The WB results further supported this trend (Figure 3F,G). These breakthroughs partially suggest that Epac‐2 activation protected the intestinal barrier in *Il*‐*10* ^−/−^ mice.

### 3.4 Epac‐2 activation did not affect Caco‐2 cell monolayers with LPS‐induced damage

**FIGURE 4:**
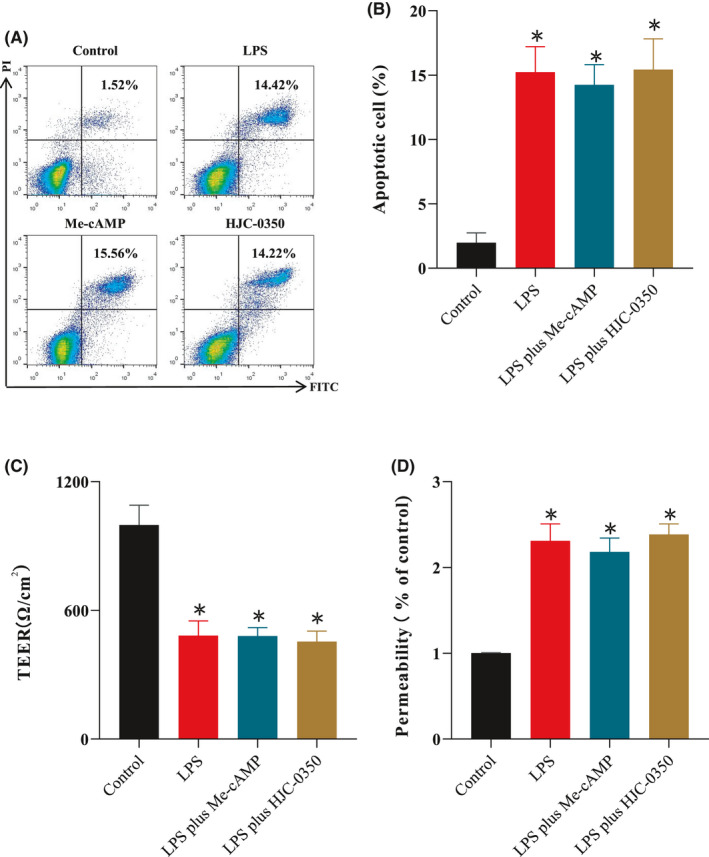
Epac‐2 activation did not affect LPS‐damaged monolayers of Caco‐2 cells. (A, B) Flow cytometry analyses of annexin V and PI in the Caco‐2 cell line for apoptosis. (C) The TEER values of Caco‐2 cell monolayers cultured alone in the control group, LPS group, Me‐cAMP group and HJC‐0350 group. (D) The percentage of FD4 transport of Caco‐2 cell monolayers cultured alone in the control group, LPS group, Me‐cAMP group and HJC‐0350 group. Caco‐2 cells were incubated for 24 h with (1) normal DMEM (control); (2) lipopolysaccharide (LPS group); (3) LPS plus Me‐cAMP group; or (4) LPS plus HJC‐0350 group. The experiments were performed 5 independent times, and the most typical result is shown. The data are presented as the means ± SD (^※^ *p* < 0.05, compared to the control group; ^#^ *p* < 0.05, compared to the LPS group)

To examine the effects of Epac‐2 on epithelial cells and macrophages, the LPS‐induced death and permeability of Caco‐2 cells were assessed under different culture conditions. After Caco‐2 cells were seeded, LPS stimulation and treatments were administered. The proportion of apoptosis in LPS‐induced Caco‐2 cells (14.42 ± 3.21%) was higher than that in the control group (1.52 ± 0.71%) but was not different from that in the Me‐cAMP group (15.56 ± 3.88%) or HJC‐0350 group (14.22 ± 3.68%, Figure 4A,B). The TEER value of the Caco‐2 cell monolayer reached 1010.02 Ω/cm^2^. The resistance in the Me‐cAMP group (469.09 Ω/cm^2^) and HJC‐0350 group (478.27 Ω/cm^2^) was similar to that in the LPS group (491.52 Ω/cm^2^, Figure 4C). Permeability was determined using FD4. The percentage of FD4 transport to the lower layer was not different among the LPS, Me‐cAMP and HJC‐0350 groups but exceeded the level of the control group by 2.3‐, 2.2‐ and 2.3‐fold, respectively (Figure 4D). These findings demonstrated that Epac‐2 activation by Me‐cAMP in Caco‐2 cells did not ameliorate LPS‐induced damage to cell monolayers.

### 3.5 Epac‐2 activation protected intestinal epithelial cells in the Caco‐2 and RAW 264.7 cell co‐culture system

**FIGURE 5:**
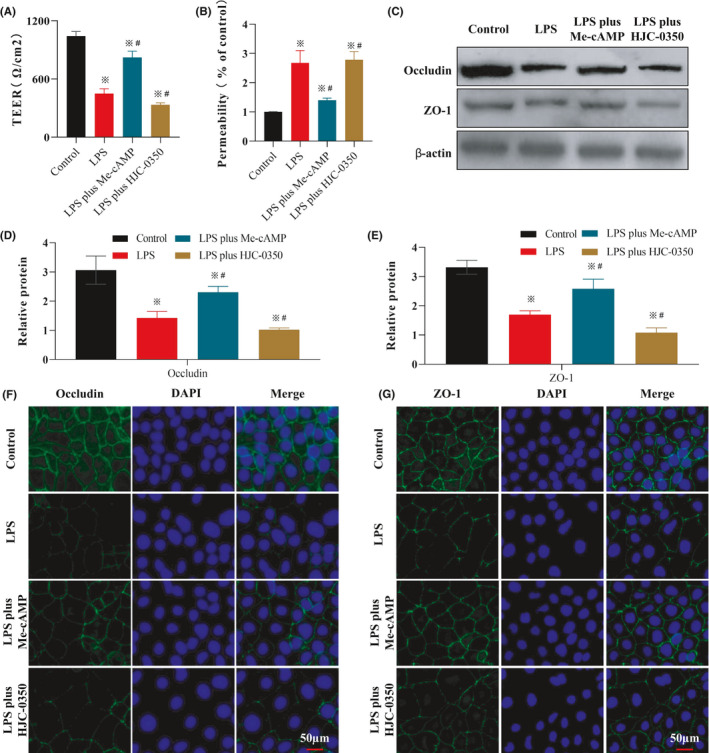
Epac‐2 activation improves LPS‐damaged Caco‐2 cell monolayers via inhibition of RAW 264.7 cell injury. (A) The TEER values of Caco‐2 cell monolayers cultured together with RAW 264.7 cells in a Transwell plate. (1) RAW 264.7 cells incubated with normal DMEM (control group); (2) RAW 264.7 cells induced with LPS for 24 h (LPS group); (3) RAW 264.7 cells pre‐treated with Me‐cAMP for 1 h, then induced with LPS (LPS plus Me‐cAMP group); (4) RAW 264.7 cells pre‐treated with HJC‐0350 group for 1 h, then induced with LPS (LPS plus HJC‐0350 group). (B) The percentage of FD4 transport of Caco‐2 cell monolayers cultured with RAW 264.7 cells in a Transwell plate. (C–E) The WB results revealed the expression of occludin and ZO‐1 in Caco‐2 cells in the co‐culture experiment. The cell supernatants of RAW 264.7 cells in the control group, LPS group, LPS plus Me‐cAMP group and LPS plus HJC‐0350 group were collected, added to the corresponding Caco‐2 cells, and incubated for 24 h. The relative proteins are shown as fold changes of protein expression in the Me‐cAMP group, the WT group and the *Il*‐*10* ^−/−^ group relative to the HJC‐0350 group. (F, G) Immunofluorescence analysis revealed that the distribution of occludin (green) and ZO‐1 (green) in Caco‐2 cells of the co‐culture experiment. DAPI (blue) was used to stain the nuclei. The experiments were performed 5 independent times, and the most typical result is shown. The data are presented as the means ± SD (^※^ *p* < 0.05, compared to the control group; ^#^ *p* < 0.05, compared to the LPS group)

Caco‐2 and RAW 264.7 cells were co‐cultured in a Transwell plate, and the TEER value of Caco‐2 cells in the LPS group (422.34 Ω/cm^2^) was less than that of cells in the control group (1047.11 Ω/cm^2^). The TEER value was increased by Me‐cAMP (836.12 Ω/cm^2^) but reduced by HJC‐0350 (337.61 Ω/cm^2^, Figure 5A). Analysis of the percentage of FD4 transport confirmed this trend, and the LPS, Me‐cAMP and HJC‐0350 groups exhibited 2.2‐, 1.4‐ and 2.8‐fold higher transport, respectively, than the control group (Figure 5B). To investigate whether Epac‐2 activation improved damage to the epithelial barrier via inhibition of the injury response of macrophages, a co‐culture experiment was performed (Figure S2). The expression levels of ZO‐1 and occludin in Caco‐2 cells in the Me‐cAMP group were significantly increased compared to the LPS‐induced Caco‐2 cells. The levels of occludin and ZO‐1 were further attenuated in the HJC‐0350 group compared to the LPS‐induced Caco‐2 cells (Figure 5C–E). The IF results further supported this trend (Figure 5F,G). These findings demonstrated that Epac‐2 activation by Me‐cAMP in RAW 264.7 cells ameliorated damage to Caco‐2 cell monolayers via inhibition of the RAW 264.7 cell response.

### 3.6 Epac‐2 suppressed the p65 and p38 signalling pathways

**FIGURE 6:**
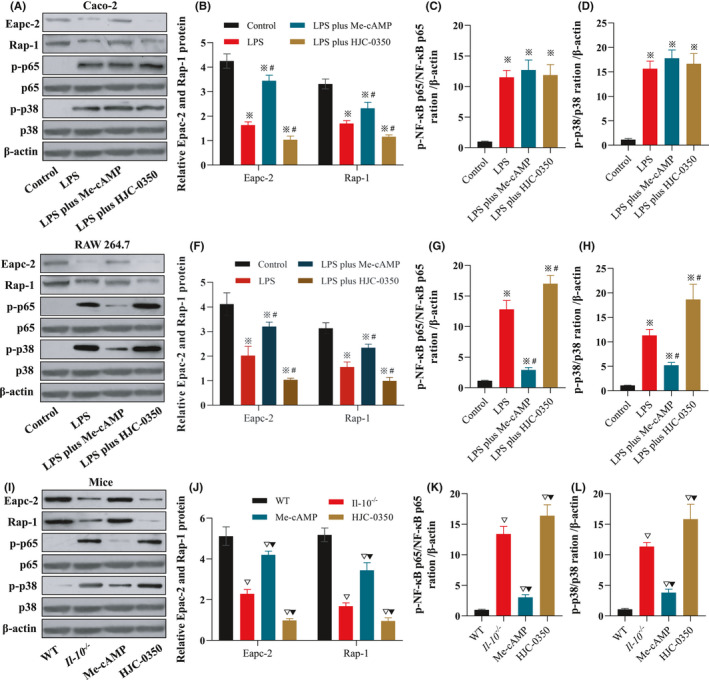
Epac‐2 suppresses damage‐induced phosphorylation of p65 and p38 signalling pathway components in macrophages by activating Rap‐1. (A–D) The WB results revealed the expression of Epac‐2/Rap‐1 signalling and the phosphorylation of p65 and p38 in LPS‐induced Caco‐2 cells. (E–H) The WB results revealed that the expression of Epac‐2/Rap‐1 signalling and the phosphorylation of p65 and p38 in LPS‐induced RAW 264.7. (I–L) WB results of the expression of Epac‐2/Rap‐1 signalling and the phosphorylation of p65 and p38 in mouse colonic mucosal tissue samples. Caco‐2 or RAW 264.7 cells were incubated for 24 h with (1) normal DMEM (control group); (2) lipopolysaccharide (LPS group); (3) LPS plus Me‐cAMP group; or (4) LPS plus HJC‐0350 group. The experiments were performed 3 or 5 independent times (*n* = 10), and the most typical results are shown. The data are presented as the means ± SD (^※^ *p* < 0.05, compared to the control group; ^#^ *p* < 0.05, compared to the LPS group; ^▽^ *p* < 0.05, compared to the WT group; ^▼^ *p* < 0.05, compared to the *Il*‐*10* ^−/−^ group)

Western blotting results indicated that the Epac‐2/Rap‐1 pathway was activated in the Me‐cAMP group compared to the LPS‐induced Caco‐2 group, but HJC‐0350 attenuated the expression of Epac‐2/Rap‐1 signalling. The phosphorylation of NF‐κB p65 and p38 MAPK in the Me‐cAMP and HJC‐0350 groups was similar to that in the LPS‐induced group (Figure 6A–D). Notably, the Epac‐2/Rap‐1 pathway was activated by Me‐cAMP and inhibited by HJC‐0350 in LPS‐induced RAW 264.7 cells. However, the phosphorylation of NF‐κB p65 and p38 MAPK was suppressed by Me‐cAMP and enhanced by HJC‐0350 compared with that of the LPS group (Figure 6E–H). Western blotting analyses of intestinal tissue samples from *Il*‐*10* ^−/−^ mice further supported these trends (Figure 6I–L). These findings partially demonstrated that Epac‐2 suppressed the phosphorylation of NF‐κB p65 and p38 MAPK in macrophages by activating Rap‐1.

## 4 DISCUSSION

To our knowledge, the present study was the first study to suggest that Epac‐2 activation ameliorated CD‐like colitis in *Il*‐*10* ^−/−^ mice. The major findings were as follows: (1) Epac‐2 improved CD‐like colitis by upregulating the expression of TJ proteins; (2) Epac‐2 protected the function of the intestinal barrier by inhibiting the inflammatory response in macrophages; and (3) the phosphorylation of NF‐κB/MAPK signalling components was attenuated by activation of Epac‐2/Rap‐1 signalling in macrophages in the bowel of *Il*‐*10* ^−/−^ mice.

The present study demonstrated that the Epac‐2 protein exerted significant protective effects against spontaneous enteritis in mice. The expression of cytokines is upregulated during the impairment of intestinal barrier function.^32^ Our surveillance data indicated that Epac‐2 decreased the inflammatory score, DAI and cytokine levels. Thus, we analysed the indicators associated with changes in intestinal barrier function and discovered that Me‐cAMP reversed the attenuation of TJ protein expression and weakened intestinal permeability in *Il*‐*10* ^−/−^ mice. Tight junction proteins are associated with intestinal barrier function, and occludin and ZO‐1 partially reflect the degree of inflammation in the intestine.^33^, ^34^ Our data suggested that the positive effect of Epac‐2 in *Il*‐*10* ^−/−^ mice was mediated by increasing the expression of TJ proteins in the colon to some extent. Disturbance of intestinal epithelial barrier function increases intestinal permeability in CD.^35^ We observed remarkable reductions in the levels of serum glucan binding and the possibility of bacterial translocation in the Me‐cAMP group. These data demonstrate that Epac‐2 has an excellent protective effect against CD.

Epac‐2‐mediated protection of the epithelial barrier was especially encouraging and confirmed the hypothesis that Epac‐2 has therapeutic effects in CD. The macrophage inflammatory response contributes to the functional disruption of the intestinal barrier.^36^, ^37^ We found that the administration of Me‐cAMP attenuated the activation of NF‐κB/MAPK signalling and induced the activation of the Epac‐2/Rap‐1 pathway in macrophages. Interestingly, phosphorylation of NF‐κB/MAPK signalling components plays a facilitatory role during the macrophage inflammatory response.^38^, ^39^ We found that Epac‐2 activation promoted functional recovery of the intestinal barrier by increasing the expression of TJ proteins and decreasing Caco‐2 cell apoptosis in the LPS‐induced cell co‐culture system, which may explain why Epac‐2 ameliorated spontaneous enteritis in *Il*‐*10* ^−/−^ mice. The protective effects of Epac‐2 on the intestinal barrier may be partially due to the suppression of NF‐κB/MAPK phosphorylation in macrophages. The NF‐κB/MAPK signalling pathway has been shown to be critical for functional recovery of the intestinal barrier.^40^

Our study still has certain limitations. For example, LPS cannot induce the apoptosis of Caco‐2 cells at low concentration (10 ng/ml), but it can induce the apoptosis at high concentrations (1 μg/ml, Figure S3). Although there was apoptosis in the LPS group, we could not determine whether it was necrosis or not, which might partly affect the reliability of the results. In addition, the results showed that Me‐cAMP increased the levels of Epac‐2, which suggests that Me‐cAMP functions via other mechanisms. Our data showed that Epac‐2 promoted functional recovery of the intestinal barrier in CD by ameliorating the inflammatory response in macrophages. However, Epac‐2 may also improve CD via other means. Attenuation of the activation of NF‐κB/MAPK signalling and increasing the levels of Rap‐1 may explain the positive role of Epac‐2 on barrier function and its mechanism of action, but other signalling pathways should also be examined because Epac‐2 may have various biological functions.^41^, ^42^

In conclusion, our study demonstrated that Epac‐2 activation ameliorated pathophysiological processes related to CD‐like symptoms in *Il*‐*10* ^−/−^ mice via suppression of cytokine expression in macrophages and increasing the levels of TJ proteins in epithelial cells. Epac‐2 may have a positive impact by decreasing the phosphorylation of NF‐κB/MAPK signalling components and increasing the levels of Rap‐1 in macrophages in the gut. These data demonstrated that Epac‐2 had a remarkable protective effect against CD and offers a novel therapeutic strategy for the treatment of CD.

## CONFLICT OF INTEREST

The authors declare no financial conflicts of interest.

## AUTHOR CONTRIBUTIONS

**Jianguo Hu:** Conceptualization (lead); funding acquisition (equal); supervision (lead); writing – review and editing (lead). **Xue Song:** Conceptualization (equal); funding acquisition (equal); methodology (equal); writing – original draft (equal). **Hexin Wen:** Conceptualization (equal); methodology (equal); validation (equal); writing – original draft (equal). **Lugen Zuo:** Data curation (equal); formal analysis (equal); funding acquisition (equal); investigation (equal). **Zhijun Geng:** Data curation (equal); formal analysis (equal); investigation (equal); visualization (equal). **Jing Nian:** Data curation (equal); formal analysis (equal); investigation (equal). **Luyao Wang:** Data curation (equal); formal analysis (equal); investigation (equal). **Zihan Zhu:** Data curation (equal); formal analysis (equal); investigation (equal). **Jing Tao:** Data curation (equal); formal analysis (equal); investigation (equal). **Yifan Jiang:** Data curation (equal); formal analysis (equal); investigation (equal). **Xiaopei Wu:** Data curation (equal); formal analysis (equal); investigation (equal). **Zhikun Wang:** Data curation (equal); formal analysis (equal); investigation (equal). **Ping Xiang:** Methodology (equal); resources (equal); visualization (equal). **Xiaofeng Zhang:** Methodology (equal); resources (equal); visualization (equal). **Hao Zhao:** Methodology (equal); validation (equal); visualization (equal). **Liang Yu:** Methodology (equal); resources (equal); visualization (equal). **Jing Li:** Methodology (equal); resources (equal); visualization (equal). **Lin Shen:** Methodology (equal); resources (equal); visualization (equal).

## Supporting information

Fig S1‐S3

## Data Availability

The data that support the findings of this study are available from the corresponding author upon reasonable request.
