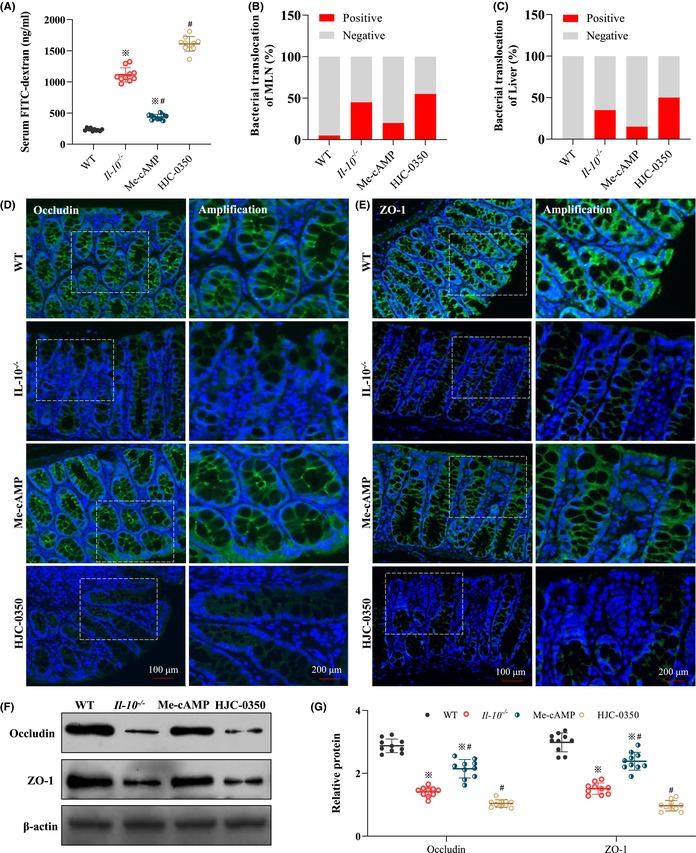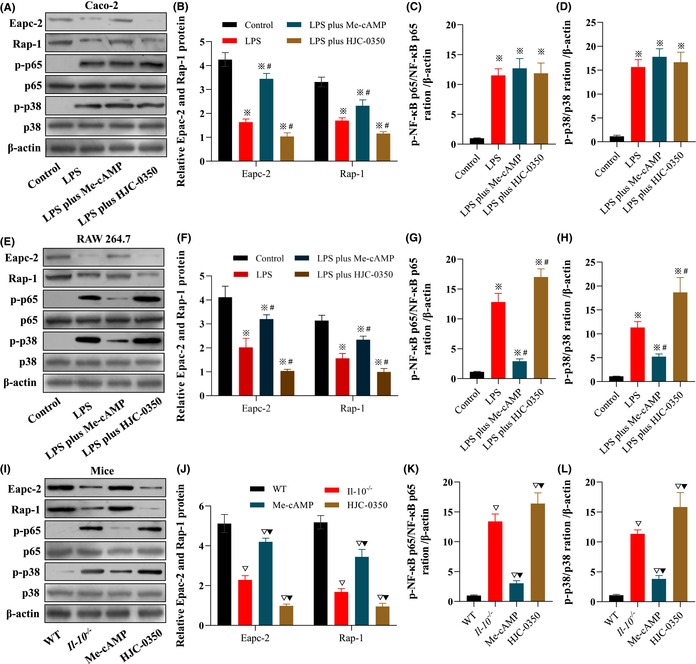# Correction to Epac‐2 ameliorates spontaneous colitis in *Il‐10* ^−/−^ mice by protecting the intestinal barrier and suppressing NF‐κB/MAPK signalling

**DOI:** 10.1111/jcmm.17885

**Published:** 2023-08-23

In Song X et al.,^1^ the β‐actin band in Figure 3F; the p65 band, p38 band and β‐actin band of Caco‐2 cells in Figure 6A; the p65 band, p38 band and β‐actin band of RAW 264.7 cells in Figure 6E; and the p65 band, p38 band and β‐actin band of mice in Figure 6I have been reused multiple times. After a review by the editor, these errors were not made by the authors. It is possible that reuse of the image was due to inadvertent errors in the formatting software or tools, or it may have been caused by technical issues in the production process of the paper. The corrected Figure 3 and Figure 6 are shown below. The authors and editor confirm that the results and conclusions of this article remain unchanged.