# Correction to ‘Epac‐2 Ameliorates Spontaneous Colitis in Il‐10^−/−^ Mice by Protecting the Intestinal Barrier and Suppressing NF‐Κb/MAPK Signalling’

**DOI:** 10.1111/jcmm.70253

**Published:** 2024-12-24

X. Song, H. Wen, L. Zuo, Z. Geng, J. Nian, L. Wang, Y. Jiang, J. Tao, Z. Zhu, X. Wu, Z. Wang, X. Zhang, L. Yu, H. Zhao, P. Xiang, J. Li, L. Shen, and J. Hu, “Epac‐2 Ameliorates Spontaneous Colitis in Il‐10^−/−^ Mice by Protecting the Intestinal Barrier and Suppressing NF‐Κb/MAPK Signalling,” *Journal of Cellular and Molecular Medicine* 26, no. 1 (2022): 216–227, https://doi.org/10.1111/jcmm.17077.

Concerns were raised by a third party regarding the apoptosis measurements in Figure 4. The main concern was the absence of a defined early apoptotic population in the plots presented in Figure 4A, which generates a reasonable doubt that the overlap between apoptotic population (early apoptosis) and the necrotic population (late apoptotic) is an artefact, and it might represent incorrect flow cytometry analyses. The authors were not able to provide the original FACS data files for Figure 4A, therefore, repeated the experiments in question to provide further clarification. The new data confirmed the same trends as observed before; therefore, the experimental results and corresponding conclusions mentioned in section 3.4 of the paper remain unaffected.

The corrected Figure 4A,B are as follows:

**Figure:**
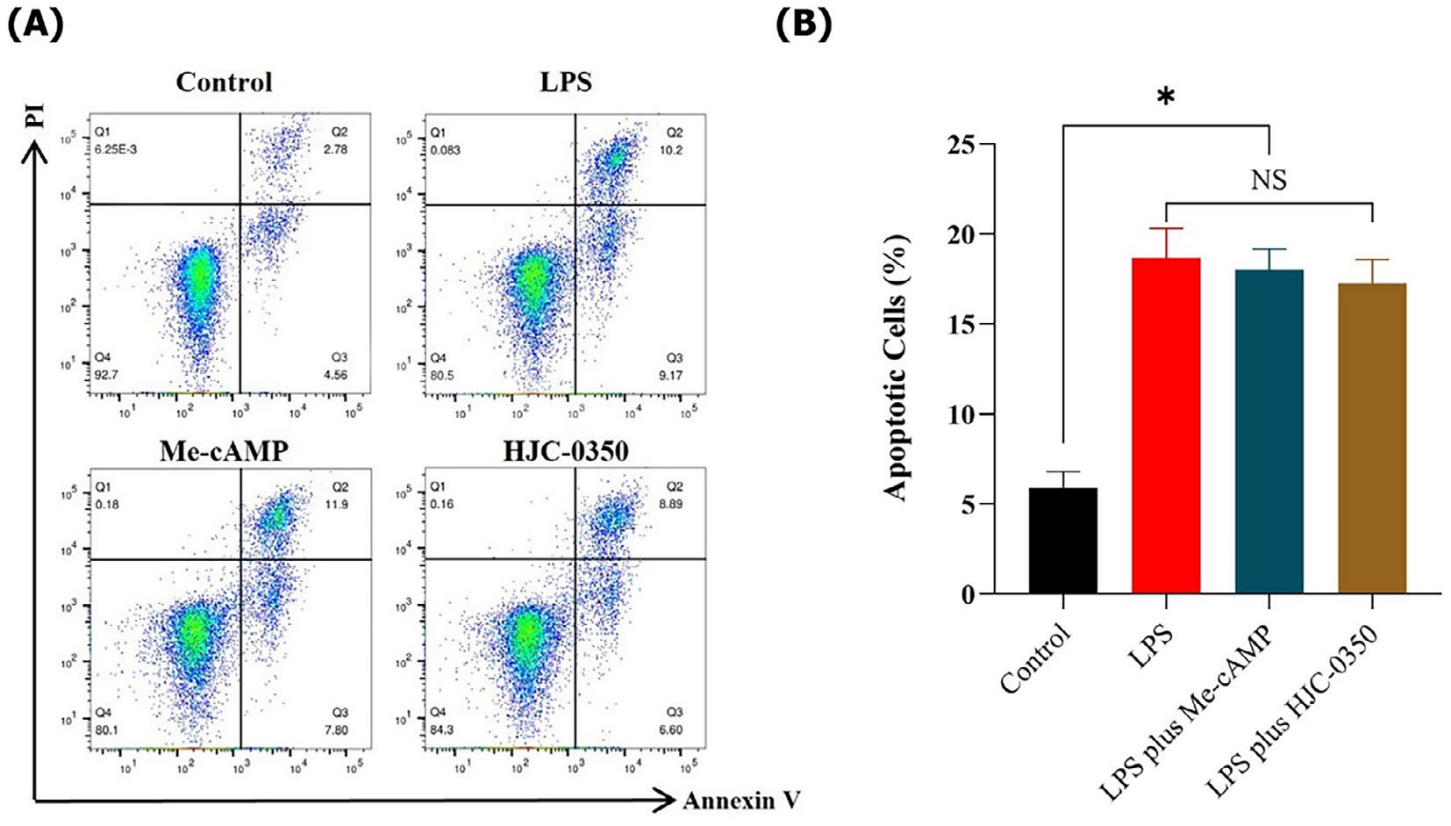
**FIGURE 4** Activation of Epac‐2 does not affect apoptosis of Caco‐2 monolayers induced by LPS, the apoptosis means early + late apoptosis. (A, B) Flow cytometry analyses of Annexin V and PI in the Caco‐2 cell line for apoptosis, the apoptosis means early + late apoptosis (Q2 + Q3).

When repeating the experiments, the authors used the Annexin V‐IF647/PI Cell Apoptosis Detection Kit, in which IF647 fluorescence is detected using the FL2 (APC) channel, and PI is detected using the FL1 (PE) channel. The compensation between the two fluorescence channels is smaller, which allows for clear cellular population and obtaining accurate early apoptotic and late apoptotic cells. For flow data collection, the authors used the BD FACSCanto flow cytometer instrument. The detailed experimental protocol of the original and repeated experiments was added to the updated Supporting Information.

For further clarification, the authors have introduced the following sentences in the fourth paragraph of the discussion section:

‘Although apoptosis was seen in the LPS group, we were unable to determine whether it was cell necrosis, and some of the necrotic cells may have been identified as late apoptotic cells, which may partially affect the reliability of the results’.

