# Supplementary material for: Epac‐2 ameliorates spontaneous colitis in Il‐10 −/− mice by protecting the intestinal barrier and suppressing NF‐κB/MAPK signalling
Source: J Cell Mol Med. 2021 Dec 3;26(1):216–27. doi: 10.1111/jcmm.17077 (PMC8742196; doi:10.1111/jcmm.17077)
Supplement: Supplementary file 1 — Fig S1‐S3 [file JCMM-26-216-s001.doc]

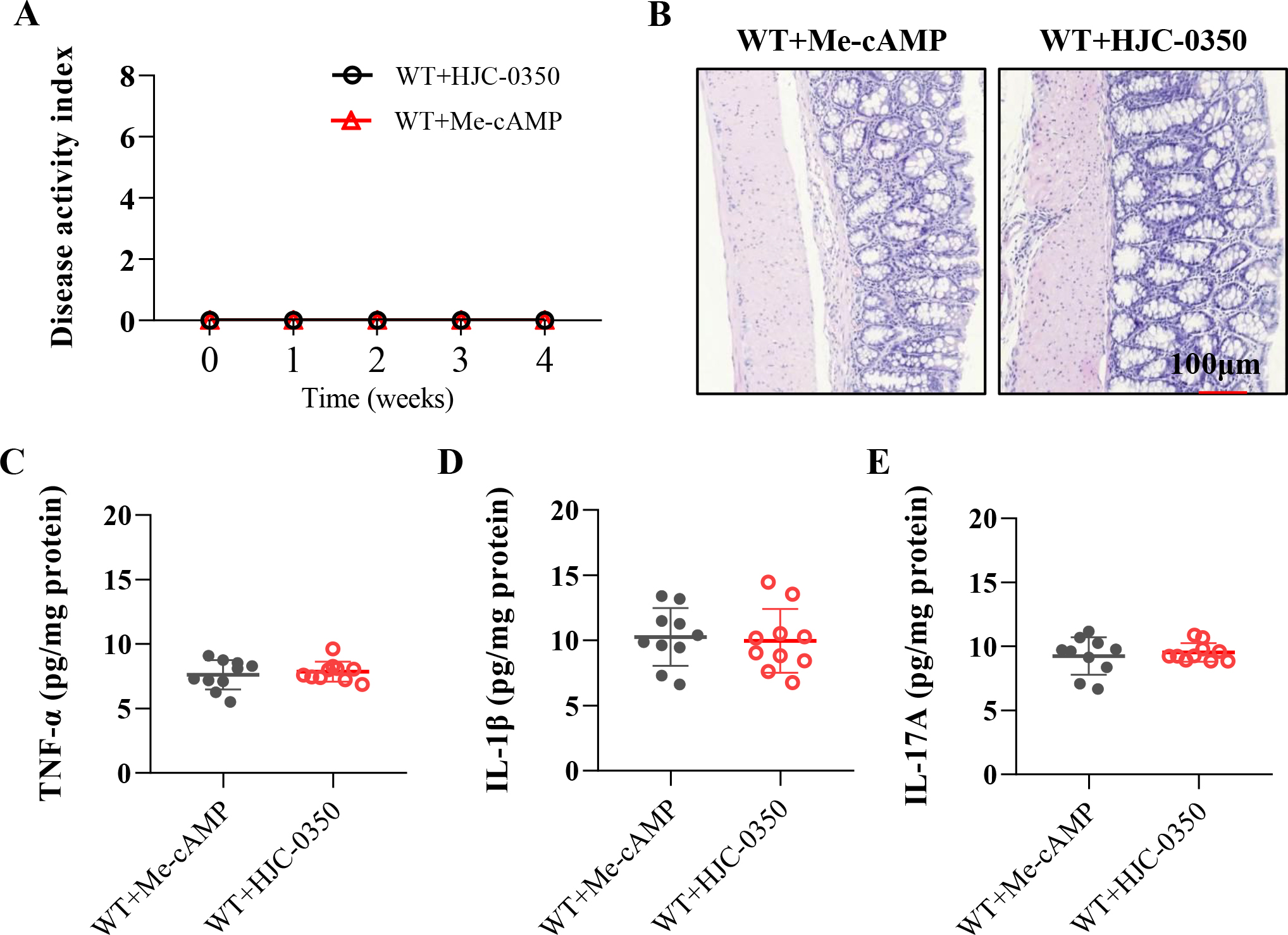


***Figure S1. Effect of Me-cAMP on colonic tissues in WT mice***

(A)The DAI scores of mice in the WT+Me-cAMP group and WT+HJC-0350 group, the DAI was obtained using a 6-point (0-5) scale, each mouse was evaluated once a week. (B) Haematoxylin and eosin (H&E) staining exhibited the histologic manifestations of colonic tissues in each group after 4 weeks of treatment. (C-E) The protein levels of TNF-α, IL-1β and IL-17A in colon collected from mice in each group. The experiments were performed 3 independent times (n=10), and the most typical result is shown. The data are presented as the means ± SD.


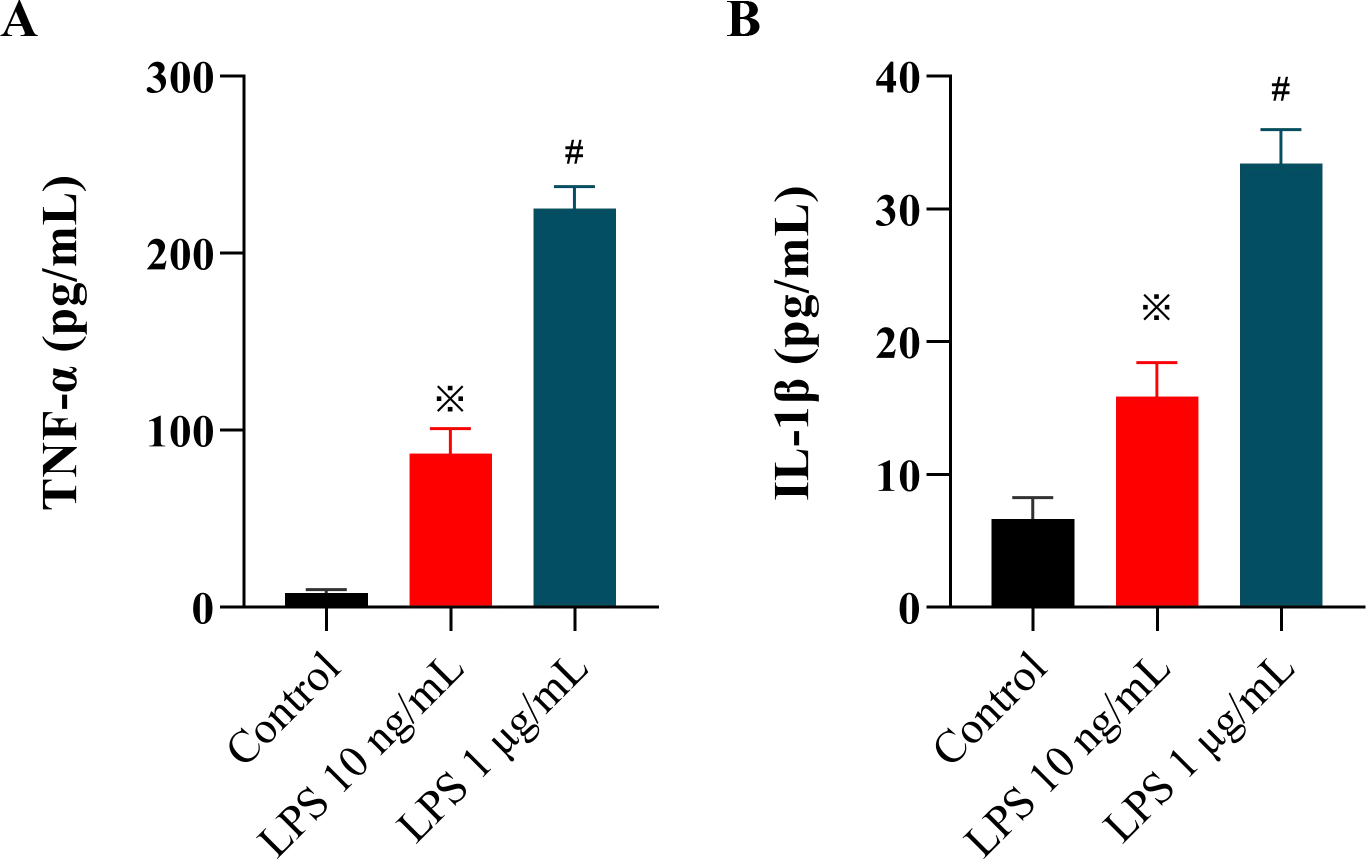


***Figure S2. Effect of LPS on RAW 264.7 cell***

(A-B) The protein levels of TNF-α and IL-1β in RAW 264.7 cell from low concentration (10 ng/ml) group and high concentrations (1 μg/ml) group. The experiments were performed 3 independent times (n=5), and the most typical result is shown. The data are presented as the means ± SD (^※^*P<*0.05, compared to the Control group; ^#^*P<*0.05, compared to the low concentration group).


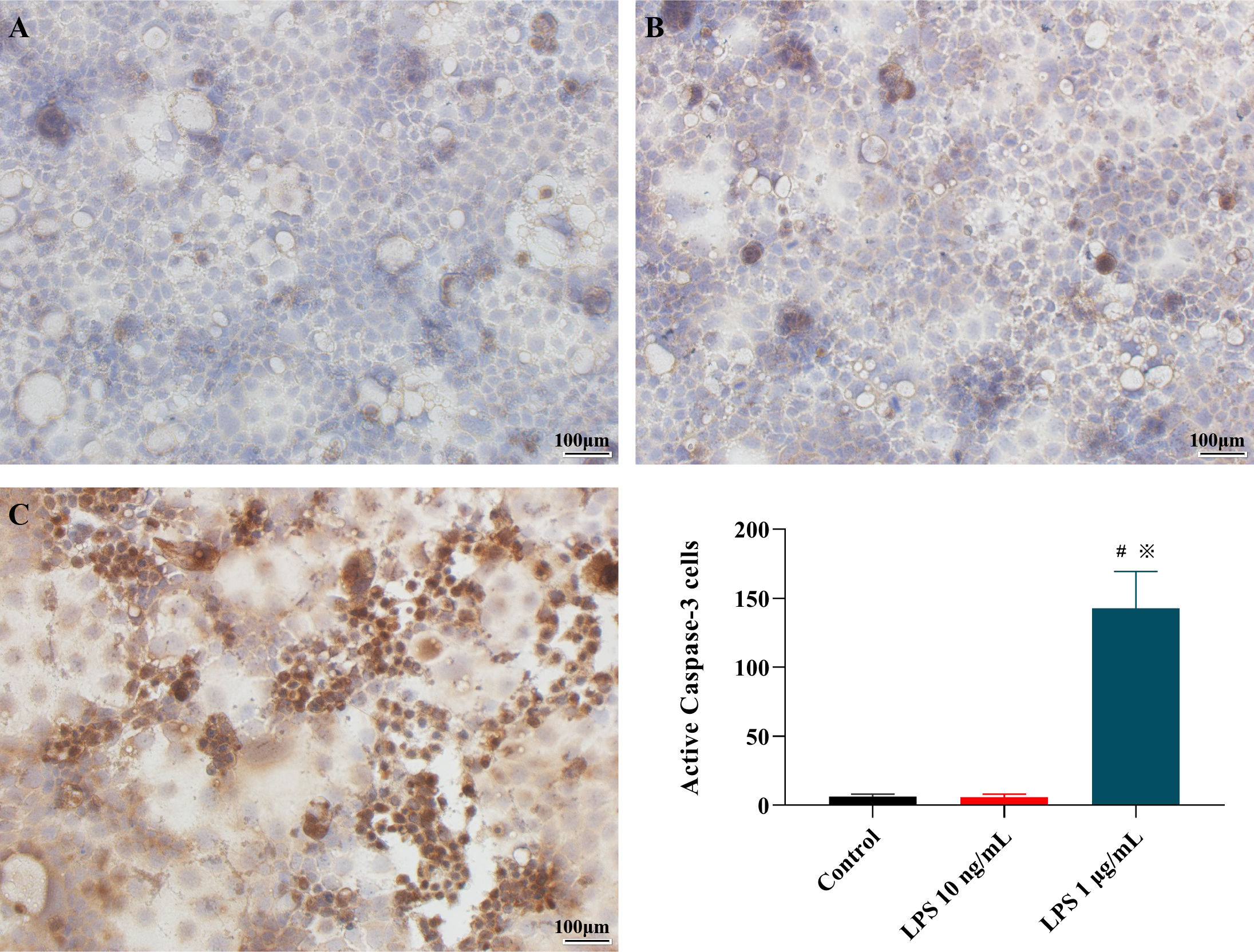


***Figure S3. LPS induced the apoptosis of Caco2 cells at higher concentration***

The expression of c-caspases3 by immunohistochemistry in Caco2 cell. (A) The cells were incubated for 24 h with normal DMEM; (B) The cells were incubated with 10 ng/ml LPS; (C) The cells were incubated with 1 μg/ml LPS. The experiments were performed 3 independent times (n=5), and the most typical result is shown. The data are presented as the means ± SD (^※^*P<*0.05, compared to the Control group; ^#^*P<*0.05, compared to the low concentration group).
